# Efficacy and safety of Xian-Lian-Jie-Du optimization decoction as an adjuvant treatment for prevention of recurrence in patients with stage IIIB/IIIC colon cancer: study protocol for a multicentre, randomized controlled trial

**DOI:** 10.1186/s12906-023-04052-2

**Published:** 2023-07-17

**Authors:** Xuechen Geng, Ziqiang Wang, Li Feng, Yanhong Gu, Renjie Wang, Qinghua Yao, Yangxian Xu, Jianyu Wu, Zhiwei Jiang, Kai Chen, Wenwei Hu, Dongxin Tang, Jiege Huo, Ling Li, Qianqian Bu, Shuoqi Zhao, Bei Zhang, Haibo Cheng

**Affiliations:** 1 The First Clinical Medical College, Jiangsu Collaborative Innovation Center of Traditional Chinese Medicine Prevention and Treatment of Tumor, Nanjing University of Chinese Medicine, Nanjing, China; 2 Department of General Surgery, Colorectal Cancer Center, West China Hospital, Sichuan University, Chengdu, China; 3 Traditional Chinese Medicine Department, National Cancer Center/Cancer Hospital, Chinese Academy of Medical Science and Peking Union Medical College, Beijing, China; 4 Department of Oncology and Cancer Rehabilitation Centre, The First Affiliated Hospital of Nanjing Medical University, Nanjing, China; 5 Department of Colorectal Surgery, Fudan University Shanghai Cancer Center, Shanghai, China; 6 Department of Integrated Chinese and Western Medicine, Institute of Basic Medicine and Cancer (IBMC), The Cancer HospitalUniversity of Chinese Academy of Sciences (Zhejiang Cancer Hospital)Chinese Academy of Sciences, Hangzhou, China; 7 Department of General Surgery, Longhua Hospital, Shanghai University of Traditional Chinese Medicine, Shanghai, China; 8 No. 2 Surgery Department, The First Affiliated Hospital of Guangzhou University of Traditional Chinese Medicine, Guangdong, China; 9 Department of General Surgery, The Affiliated Hospital of Nanjing University of Chinese Medicine, Jiangsu Province Hospital of Chinese Medicine, Nanjing, China; 10 Department of Oncology, The First Affiliated Hospital of Soochow University, Suzhou, China; 11 Department of Oncology, The Third Affiliated Hospital of Soochow University, Changzhou, China; 12 Clinical Medical Research Center, The First Affiliated Hospital of Guizhou University of Traditional Chinese Medicine, Guiyang, China; 13 Affiliated Hospital of Integrated Traditional Chinese and Western Medicine, Nanjing University of Chinese Medicine, Jiangsu Province Academy of Traditional Chinese Medicine, Nanjing, China; 14 Chinese Evidence-Based Medicine Center, NMPA Key Laboratory for Real World Data Research and Evaluation in Hainan, Sichuan Center of Technology Innovation for Real World Data, West China Hospital, Sichuan University, Chengdu, China; 15 State Key Laboratory of Oncology in South China, Sun Yat-Sen University Cancer Center, Collaborative Innovation Center of Cancer Medicine, Guangzhou, China; 16 Department of TCM, Sun Yat-Sen University Cancer Center, Guangzhou, China

**Keywords:** Xian-Lian-Jie-Du optimization decoction, Colon cancer, Chinese herbal medicine, Randomized controlled trial, Protocol

## Abstract

**Introduction:**

Colon cancer remains one of the most prevalent cancers worldwide. Unfortunately, there are no recognized and effective therapeutic strategies to prevent tumor recurrence after radical resection and chemotherapy, and the disease-free survival (DFS) in patients with stage IIIB or IIIC disease remains unsatisfactory. Xian-Lian-Jie-Du optimization decoction (XLJDOD) is a Chinese herbal medicine (CHM) empirical prescription, which has been validated experimentally and clinically that could inhibit the progression of colorectal cancer and ameliorate the symptoms. The purpose of this study is to evaluate the efficacy and safety of XLJDOD in prevention of recurrence of colon cancer.

**Methods:**

This study is a multi-center, double-blind, randomized, placebo-controlled trial conducted at 13 hospitals of China. Following the completion of surgery and adjuvant 5- fluorouracil-based chemotherapy, a total of 730 subjects with stage IIIB or IIIC colon cancer will be randomized in a 1:1 ratio to an intervention group (*n* = 365; XLJDOD compound granule) and a control group (*n* = 365; Placebo). Patients will receive 6-month treatments and be followed up with 3 monthly assessments for 2 years. The primary outcome is 2-year DFS rate and the secondary outcomes are 1, 2-year relapse rate (RR), overall survival (OS) and quality of life (QoL). Safety outcomes such as adverse events will be also assessed. A small number of subgroup analysis will be carried out to explore the heterogeneity of effects of XLJDOD.

**Discussion:**

The outcomes from this randomized controlled trial will provide objective evidences to evaluate XLJDOD’s role as an adjuvant treatment in colon cancer.

**Trial registration:**

www.ClinicalTrials.gov, identifier: NCT05709249. Registered on 31 Jan 2023.

**Supplementary Information:**

The online version contains supplementary material available at 10.1186/s12906-023-04052-2.

## Introduction

According to estimates from the International Agency for Research on Cancer (IARC), colorectal cancer has the third highest incidence and the second highest mortality worldwide in 2020, in which the occurrence and death risk of colon cancer is about half higher than that of rectal cancer. The prevention and control situation is still grim with about 1.15 million new cases and about 0.58 million deaths due to colon cancer in 2020 [1]. For those without evidence of metastatic disease, surgical resection is the main treatment modality, and it is commonly followed by adjuvant chemotherapy, which can bring survival benefits for patients with colon cancer [2, 3]. Unfortunately, approximately 1/3 of patients have stage III disease at diagnosis because of lymph node involvement [4–6], around 40% of them relapse even after surgery and adjuvant chemotherapy [7, 8].

The main cause of death after radical resection for colon cancer is tumor recurrence [9]. The study found that the risk of recurrence is highest in the first 2 years and that the risk of death peaks about 2 years after treatment [10]. Stage III colon cancer comprises a highly heterogeneous group of patients, with a 2-year rate of disease-free survival (DFS) of about 91% (IIIA), 79% (IIIB) and 60% (IIIC), respectively [11]. In addition, high levels of symptom burden-fatigue, insomnia, distress, constipation, and diarrhoea-is often expected shortly after treatment, which can result in significant limitations in daily activities and overall impairment in health-related quality of life (QoL) [12]. However, there lacks a recognized and effective therapeutic strategy in modern medicine to prevent recurrence after radical resection and chemotherapy for patients with stage IIIB or IIIC colon cancer. Additional strategies are required urgently in consequence.

Traditional Chinese medicine (TCM) is an important part of comprehensive treatment of cancer, which has been proven to prevent recurrence of many tumors [13–15]. In the past few years, traditional Chinese herbal medicine (CHM) has gained more and more attention in the field of colorectal cancer due to its favorable safety and efficacy profiles. A great quantity of Chinese patients with colon cancer seek help from TCM during or after standard first-line therapy. Prospective cohort studies confirmed that long duration of TCM herbal use was associated with improved disease-free survival outcomes in patients with stage II and III colorectal cancer [16–18]. A retrospective cohort study indicated that adherence to medication of TCM after surgery could significantly improve disease-free survival in colorectal patients with stage III disease [19]. Furthermore, a randomized controlled trial of 370 patients showed that Chinese herbal formula composed of seven herbs (PRM1201) in combination with adjuvant chemotherapy in the treatment of stage III colon cancer could effectively increase the 3-year DFS rate and improve their life quality [20].

On the basis of inheriting the theory of cancer toxin [21], Xian-Lian-Jie-Du optimization decoction (XLJDOD, original name: Xian-Lian-Jie-Du decoction) was developed by Professor Cheng Haibo to inhibit the progression of colorectal cancer. The herbal drugs of XLJDOD is shown in Table 1. A preliminary randomized controlled clinical study with 60 subjects revealed that XLJDOD combined with capecitabine plus oxaliplatin (CAPOX) could promote cellular immune function of patients with advanced colorectal cancer, enhance their QoL and tolerance to chemotherapy [22].

**Table 1:** Standard formulation of Xian-Lian-Jie-Du Optimization Decoction (XLJDOD)

Pinyin name	Latin name	Pharmacological effects	Doses, g
Huangqi	*Astragali Radix*	regulate immune function, affect cell cycle, promote apoptosis and neovascularization, inhibit inflammation response and reverse multi-drug resistance [23]	30
Baizhu	*Atractylodis Macrocephalae Rhizoma*	induct tumor cell apoptosis, inhibit tumor stem cell characteristics, regulate metabolic activity, inhibit invasion and metastasis, reverse chemoresistance, overcome tumor immune evasion [24]	12
Sanleng	*Sparganii Rhizoma*	one of the most frequently used herb pairs: inhibit cell proliferation, migration, invasion and stemnes, cycle arrest, regulate immunity, anti-angiogenesis [25, 26]	9
Ezhu	*Curcumae Rhizoma*		9
Yiyiren	*Coicis Semen*	promote apoptosis, reduce expression of tumor related genes, suppress chronic inflammatory microenvironment, enhance immune function [27]	15
Huanglian	*Coptidis Rhizoma*	induct apoptosis, anti-inflammation, modulate gut microbiota, regulate signal transduction, inhibit metastasis, inhibit autophagy [28]	3
Kushen	*Sophorae Flavescentis Radix*	induce cell cycle arrest, inhibit angiogenesis, induce cell differentiation, inhibit tumor metastasis and invasion, reverse multidrug resistance [29]	6
Xianhecao	*Agrimoniae Herba*	promote apoptosis and necrosis, block cell cycle, inhibit migration and invasion,enhance immune response [30]	12

However, a high-quality randomized controlled trial with sufficient sample sizes is still required urgently to prove the long-term efficacy and safety of XLJDOD in individuals with colon cancer, especially those with stage IIIB or IIIC disease who are at high risk of recurrence. Therefore, we designed such a randomized controlled trial aimed at evaluating the utility of XLJDOD as an adjuvant treatment in preventing postoperative recurrence, prolonging survival and improving QoL of people with stage IIIB or IIIC colon cancer. The purpose of this study is to present the methodologies and details of the protocol.

## Methods and design

### Study design

**Fig. 1:**
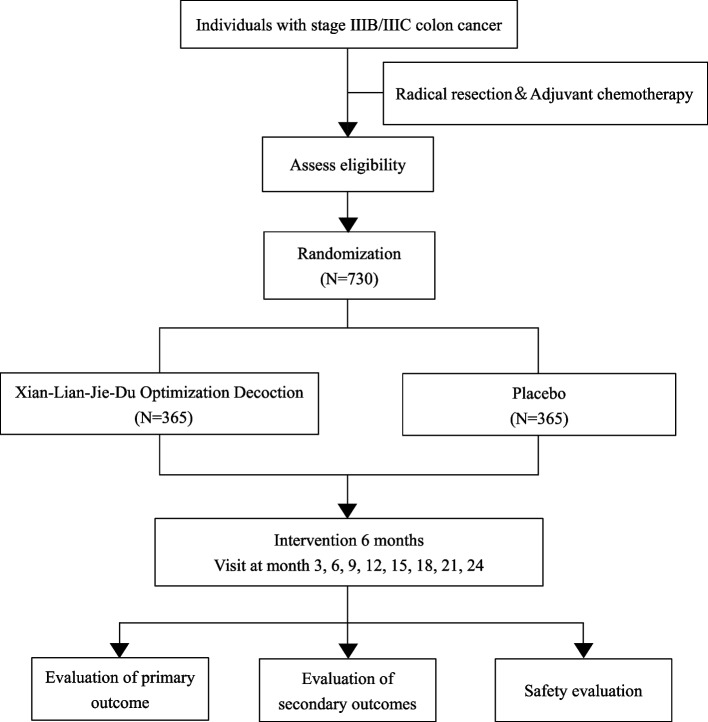
The flow diagram of this study

This study is designed as a multicentre, randomized, double-blind, parallel-arm, placebo-controlled trial. Participants will be recruited from following 13 centers, including Sun Yat-sen University Cancer Center, Cancer Hospital Chinese Academy of Medical Sciences, Fudan University Shanghai Cancer Center, West China Hospital of Sichuan University, Jiangsu Province Hospital, Zhejiang Cancer Hospital, Jiangsu Province Hospital of Chinese Medicine, LongHua Hospital Shanghai University of Traditional Chinese Medicine, The First Affiliated Hospital of Guangzhou University of Chinese Medicine, Jiangsu Province Hospital on Integration of Chinese and Western Medicine, The First Affiliated Hospital of Guizhou University of Traditional Chinese Medicine, The First Affiliated Hospital of Soochow University, and The First People’s Hospital of Changzhou. Recruitment performs simultaneously in out-patient clinics and wards. This trial was registered with ClinicalTrials.gov (NCT05709249). This protocol has been developed in accordance with the Standard Protocol Items: Recommendations for Interventional Trials (SPIRIT) [31], and the new extension of the Consolidated Standards of Reporting Trials (CONSORT) for CHM [32] to ensure appropriate high-quality methodology and strict quality control. The clinical study will be conducted for 36 months, from December, 2022 to December, 2025. The flow diagram of this study is depicted in Fig. 1.

### Inclusion criteria

- 1. Colon carcinoma entirely lying above the peritoneal reflection confirmed by pathology.
- 2. Completion of surgical resection of tumors with negative margins (R0 resection) and at least 3 months of adjuvant chemotherapy based on 5-fluorouracil (5-FU). *
  *4 cycles of CAPOX (capecitabine and oxaliplatin), 6 cycles of FOLFOX (fluorouracil, leucovorin, and oxaliplatin) or 4 cycles of single agent-5-FU, etc.
- 3. Within 3 months after the completion of adjuvant chemotherapy.
- 4. Patients with Stage IIIB or IIIC disease.*
  *IIIB: T3-T4aN1/N1cM0, T2-T3N2aM0 and T1-T2N2bM0, IIIC: T4aN2aM0, T3-T4aN2bM0 and T4bN1-N2M0, as defined by the American Joint Committee on Cancer (AJCC) 8th edition) [6].
- 5. Aged 18–80 years, men or women.
- 6. Eastern Cooperative Oncology Group (ECOG) performance status of 0–2.
- 7. With no radiographic evidence of tumor recurrence.
- 8. Sign the informed consent form.

### Exclusion criteria

- 1. Presence of other malignancies in the past 5 years except curatively treated basal cell carcinoma or cervical carcinoma in situ.
- 2. Besides adjuvant chemotherapy, other adjuvant therapy such as radiotherapy, targeted therapy and immunotherapy has been used to treat colon cancer.
- 3. Antitumor Chinese patent medicine and decoction have been used for more than 3 months after surgery or within 1 month before enrollment.
- 4. Patients with severe comorbidities such as cardiovascular, cerebrovascular, renal, hepatic, hematopoietic system and other severe primary diseases.
- 5. Allergic to the ingredients of XLJDOD.
- 6. Any condition that is unstable or can jeopardize the safety of the patients and their compliance to the study, including pregnancy, plan to be pregnant, lactation and psychiatric disorders (schizophrenia, depression, and obsessive-compulsive disorder, etc.).
- 7. Suspected or confirmed history of alcohol and drug abuse.
- 8. Patients with other conditions considered by the investigator should not participate in the study.
- 9. Patients who have recently participated in or are currently participating in other clinical trials of drugs.

### Withdrawal criteria

The investigator can decide to withdraw a subject from this study in the following circumstances:

- 1. Patients with anaphylaxis or serious adverse events (SAEs) who require the discontinuation of treatment.
- 2. Patients failure to comply with medication protocol (e.g., taking less than 80% or more than 120% of the prescribed dosage, adding other treatment drugs without following the investigator’s guidance).
- 3. Patients who break blind procedure for a variety of reasons.

The patients can discontinue their participation in the study at any time for any reason without any consequences if they are unwilling or impossible to proceed. Lost to follow-up patients who are not explicitly asked to withdraw from the trial but no longer receive experimental drug or undergo testing are also considered as withdrawn.

### Intervention measures

#### Intervention group

Subjects in the intervention group will be treated with XLJDOD compound granule. Treatment will begin within 3 months after standard adjuvant chemotherapy and compliance will be continuously monitored. XLJDOD will be taken twice a day, infused with warm water, 1 h after lunch and dinner. One course of treatment will take 28 days in 1 month, and 2- to 3-day rest. Treatment will continue for 6 courses.

#### Control group

Subjects in the intervention group will be treated with placebo (XLJDOD mimetic agent). Placebo granules contains 2% XLJDOD granules, and the remaining ingredients are 86.13% maltodextrin, 10% lactose, 1.28% caramel pigment, 0.08% lemon yellow pigment, 0.01% sunset yellow pigment, and 0.50% bitters. The course of placebo in control group will be in accordance with that of XLJDOD in intervention group. After the treatment, the packaging will be returned to the investigators.

#### Drug combination

All drug treatment received by subjects during the trial including the baseline should be recorded in detail in the case report form (CRF). Necessary treatment under the guidance of the clinician due to other diseases or symptoms is allowed, however, Chinese patent medicine and decoction with antitumor effects should be prohibited before the termination of the trial.

### Study procedure

**Fig. 2:**
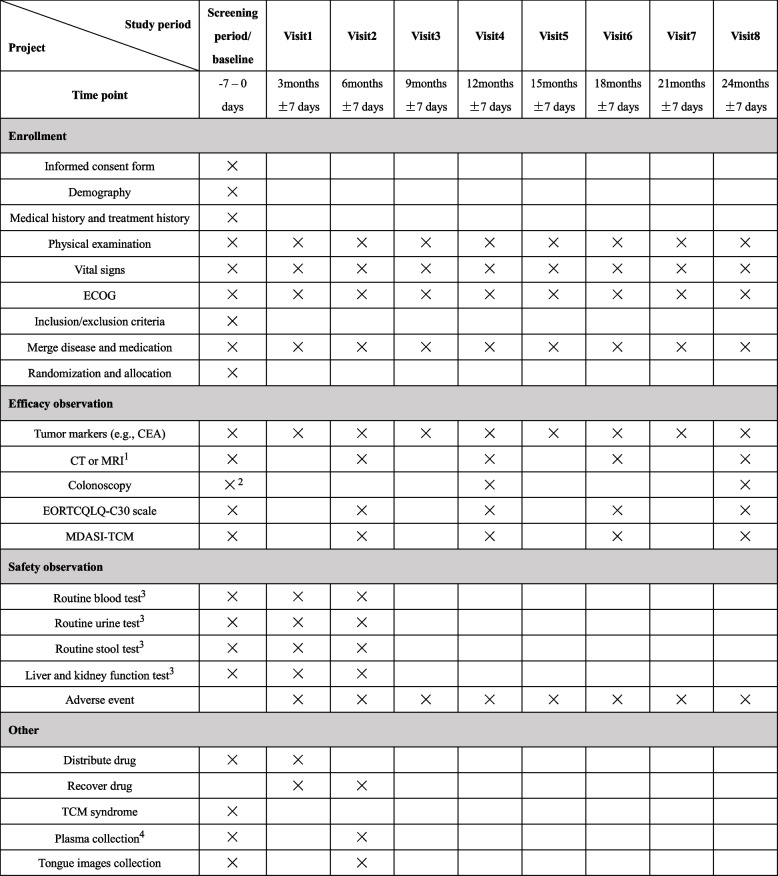
Clinical Study Schedule Form. 1 Including chest, abdomen, and pelvis computed tomography (CT) or enhanced CT scan, patients with conditions can undergo pelvis magnetic resonance imaging (MRI) scan; At baseline, patients can provide imaging reports within 3 months. 2 Colonoscopy report within 3 months is only required for patients without preoperative colonoscopy due to obstructing lesion. 3 Safety data will be reviewed every 3 months during treatment, and reviewed in accordance with the guidelines and clinician recommendations after medication intervention. 4 Plasma will be collected before and after medication and placed in –80 °C refrigerator

The duration of this study will be 3 years, with 1 year of enrollment and a minimum follow-up period of 2 years. The specific measurements and time points for data collection of this study are outlined in Fig. 2, which must be followed as accurately as possible. Treatment will be started within 7 days of the randomization and last for 6 months. Eligible patients will be followed up every 3 months until death or the end of this study. The researcher should obtain written informed consent before conducting study-specific procedures for patients enrolled in the trial.

### Outcomes

#### Primary outcome

Two-year disease-free survival (DFS) rate: DFS is defined as the time from the date of randomization to the date of disease recurrence, second primary cancer (colon cancer or other cancer), or death from any cause, whichever occurred first. 2-year DFS rate is defined as the percentage of patients alive without disease recurrence at 2 years measured from the randomization date. Disease recurrence includes any condition of the following—unequivocal radiological evidence of colon cancer locoregional recurrence or distant metastases, positive histology or cytology (e.g. peritoneal or pleural cytology), colonoscopic evidence of locoregional cancer recurrence at the anastomotic site and its adjacent areas.

#### Secondary outcomes

One, two-year relapse rate (RR): RR is defined as the fraction of followed patients who have disease recurrence. All disease recurrences and deaths from colon cancer are events. Second primary same cancers and other primary cancers will be ignored.

Overall survival (OS): OS is measured from the date of enrollment to the date of death, irrespective of cause.

Quality of life (QoL):

1. Changes in total score on the EORTCQLQ-C30 Scale: The changes of EORTCQLQ-C30 score will be compared in the two groups prior to and following XLJDOD compound granule administration. Considering that some items of EORTC QLQ-CR29 scale are targeted at colostomy patients with rectal cancer, we select the EORTCQLQ-C30 Scale. It is a universal scale for all patients with malignant tumors with a total score of 126, ranging from 30 (least severe) to 126 (most severe). Measurement will be performed at baseline and month 6, 12 ,18 ,24.

2. Changes in total score on the MD Anderson Symptom Inventory for Traditional Chinese Medicine (MDASI-TCM): Compared with the baseline period, the changes in MDASI-TCM score at month 6, 12 ,18 ,24 will be measured. The scale is divided into two parts. The first part mainly assesses the severity of 20 common clinical symptoms and TCM-related symptoms of patients with cancer in the past 24 hours. The second part is the evaluation scale of the interference of the above symptoms with daily functioning [33]. It has a total of 26 items, each with a score of 0–10, leading to a total score of 0–260.

#### Safety outcomes

- 1. Prevalence of abnormal vital signs (pulse, respiration, blood pressure and body temperature). Measurement will be performed at baseline and every 3 months within 2 years of follow-up.
- 2. Prevalence of abnormal blood routine, urine routine, stool routine, liver and kidney function tests. Measurement will be performed at baseline and every 3 months during treatment, and performed in accordance with the guidelines and clinician recommendations after medication intervention.
- 3. Other adverse events (AEs) and serious adverse events (SAEs). All adverse events should be recorded and graded in Adverse Event Form throughout the study. At the same time, the relationship between the adverse event and XLJDOD compound granule should be evaluated, and the treatment process and outcome should be recorded in detail until the laboratory examination returns to normal and the symptoms and signs disappear. When a severe adverse event occurs, immediate measures should be taken to protect the safety of the patients. Researchers must complete the Serious Adverse Event Report Form and report to the research center, the main research medical ethics committee, and the primary sponsor within 24 hours.

#### Exploratory outcomes

Plasma samples will be collected before and after medication and will be used to establish a biological sample bank, which may help to promote the research program and explore the therapeutic mechanism of XLJDOD in preventing colon cancer recurrence. At the same time, tongue images (the colour, size and shape of the tongue and the colour, thickness and moisture content of the tongue coating) will be extracted by the Chinese medicine artificial intelligence health status recognition system to investigate the value of tongue images in the prediction of colon cancer recurrence.

### Randomization and allocation concealment

In this trial, an online centralized randomization system called Interactive Web Response System (IWRS) will perform the block randomization with a 1:1 ratio, permutation block with variable sizes of four, six, eight, or twelve, stratified by the study center. After informed consent, the investigators will log into IWRS to acquire a unique identification code and random number of each participant for participant identification and treatment assignment. IWRS will ensure the allocation concealment. It will only offer a random number to the investigator, and none of the investigators, participants, and outcome evaluators will know from random numbers whether they represent the intervention group or the control group. In case of SAEs that may be associated with experimental drugs, including death, allergic reactions, intolerable severe gastrointestinal reactions, liver and kidney insufficiency, etc., the allocated intervention of participant could be revealed by investigator in the IWRS.

### Blinding

A double-blind design was adopted in this study. Investigational drugs are XLJDOD compound granule and XLJDOD mimetic agent, which are produced, packaged and provided by Tianjiang Pharmaceutical Group Co. Ltd., Wuxi, China. Mimetic agent is indistinguishable from XLJDOD compound granule in shape, size, taste, color, package, and Lot number. Each medication bag will be labelled with a unique identity number, which corresponds to the random number obtained by IWRS. Researchers, participants, statisticians, and data administrators will be blind to the treatment allocation plans throughout the trial in order to reduce the bias to the greatest extent.

### Data collection and monitoring

All participant data will be recorded on the written case report forms (CRFs), and then dual-input into and managed in form of an electronic case report form (eCRF) through an Electronic Data Capture (EDC) system. Each trail site should designate a person who is strictly trained to be responsible for the preliminary examination of the CRFs and inputting the data into eCRFs. Participants will be contacted periodically to obtain follow-up data. The EDC system will check the data automatically and data managers will review the eCRFs regularly. Suspicious data will be filled in the query list and answered by investigators. In this study, monitors with medical background will ensure the progress of trial is conducted, recorded, and reported in accordance with the protocol, and Standard Operating Procedures (SOPs). The number of site visits should meet the needs of clinical research quality control. After each site visit and/or other trial-related communication, a written report from the monitor to the sponsor is requisite.

### Sample size calculation

The primary Outcome of this study is the rate of 2-year DFS in stage IIIB or IIIC colon cancer. Based on the literature and previous study, it is assumed that the 2-year DFS rate of control group is 0.70 (70%) and of the intervention group is 0.80 (80%). Taking a two-sided significance level of 5% and power of 80% (α = 0.05, β = 0.10), the intervention group and the control group are allocated at a ratio of 1:1. Considering a dropout rate of 20%, the sample size is not less than 364 cases in each group. Therefore, a total sample size of 730 cases is set up in this study (*n* = 365 in each group).

### Statistical analysis

#### Selection of statistical analysis data set

##### Full Analysis Set (FAS)

It refers to the modified subject set that is as close as possible to the principle of Intention-to-treat (ITT) analysis (primary analysis should include all randomized subjects). The data set is obtained after all randomized subjects are eliminated by the minimum and reasonable method.

##### Per-protocol Set (PPS)

PPS comprises subjects who complete the 6 courses of treatment without serious protocol violations. Meanwhile, the baseline variables of the subjects are complete, and primary variables can be measured.

##### Safety set (SS)

SS is defined as the data set composed of all subjects who receive at least one dose of XLJDOD compound granule after randomization.

In this study, the FAS data set is the primary analysis set. In addition, a per-protocol analysis will also be performed as a sensitivity analysis.

#### Content and method of statistical analysis

The statistical analysis plan will be developed by an independent statistician who does not participate in the study in consultation with the principal researchers. SAS 9.4 will be employed for statistical analysis. The content will cover actual number of subjects enrolled in both groups, shedding and exclusion of cases, demographics and other baseline characteristics, compliance, efficacy analysis, and safety analysis.

The description of qualitative data will use frequency table, percentage or proportion. Quantitative data will be expressed by mean, standard deviation, median (P25, P75) and minimum/maximum values.

The Log-rank test will be used as the primary analysis for comparison of 2-year DFS rate in two groups, and the Cox regression will be used to adjust for covariates (such as study centers) as sensitivity analysis. Pre-determined subgroup analysis factors include sex, age (< 65 yr versus ≥ 65 yr), tumor location (left colon versus right colon), tumor pathological stage (IIIB versus IIIC), T classification (T1, T2, or T3 versus T4), N classification (N1 versus N2), the presence or absence of venous invasion. The comparison of 1,2-year RR and median OS will be performed by Log-rank test. The QoL score at different time points will be analyzed using mixed effect model and adjusting for study centers. Safety outcomes will be evaluated by summarizing the number of abnormal subjects and comparing the rate between groups. All data statistics will be analyzed by using a two-sided test, and the test statistics and their corresponding *p*-values will be given, with a significance level setting at *p* < 0.05.

## Discussion

Colorectal cancer is the second most common malignant tumor in China, with a continuous increasing trend for incidence and mortality rates [34]. 3–6 months of adjuvant 5-FU-based chemotherapy is standard for patients with stage III colon cancer after completion of surgery, and then followed by surveillance. However, there remains room for improvement in DFS of stage IIIB or IIIC colon cancer patients with high risk of recurrence. Thus, in addition to adequate surveillance, novel therapeutic strategies after adjuvant chemotherapy are required to achieve more favorable clinical efficacy.

Although many studies [35–39] have demonstrated that the low-dose use of NSAIDs, in particular Aspirin, is associated with the decreased risk of postoperative recurrence and death of colon cancer, this was not confirmed in a large randomized controlled trial [40]. Additionally, increasing studies have examined the association between vitamin D [41–43], metformin [44–47] and the recurrence and mortality of colon cancer, but generated conflicting conclusions. In the meantime, the side effects hamper the acceptance of these strategies as adjuvant agents in colon cancer.

TCM has the advantages of reliable efficacy and low treatment cost. It has been widely used in the treatment of different malignant tumors. Surveys indicate that approximately 80 percent of patients in China had used TCM, particularly CHM, during cancer treatment [48, 49]. Mounting evidence, meanwhile, suggests that Chinese medicine prescriptions have significant effect of colon cancer progression control [16, 20, 50–53]. XLJDOD is an empirical prescription used as an adjuvant treatment of colorectal cancer for more than 10 years in China. Basic biological research confirms that XLJDOD effectively inhibits tumor cell proliferation and migration, modulates tumor microenvironment, regulates the function of immune cells [54–56].

The goal of this large sample size, multicentre randomized controlled trial is to evaluate the efficacy and safety of XLJDOD as an adjuvant treatment for prevention of stage IIIB or IIIC colon cancer recurrence and to evaluate whether it can prolong survival and promote QoL. Furthermore, if the trial proves that taking XLJDOD after adjuvant chemotherapy is more potent and efficient than Placebo in reducing the risk of tumor recurrence, we will explore the mechanism of XLJDOD in the treatment of colon cancer by the plasma collected before and after the intervention.

However, this study has certain limitations. First of all, the duration of CHM therapy is relatively limited. This is due to the consideration that CHM therapy use for at least 6 months can be regarded as an effective high exposure [57], and longer duration of CHM use can lead to decreased compliance and increased rate of lost-to-follow-up. Secondly, the duration of follow-up for DFS and OS is relatively insufficient. In this study, we will primarily assess the efficacy of XLJDOD in the first 2 years after adjuvant therapy. If effective, the follow-up will be continued beyond the end of the study.

To our best knowledge, this trial represents the first attempt to evaluate CHM as an adjuvant agent after completion of surgery and standard adjuvant chemotherapy in stage IIIB or IIIC colon cancer. If shown to be beneficial, the results will provide a treatment strategy of TCM, which can be extensively and successfully used in clinical practice.

## Trial status

The protocol version is 2.0 and the date is November 29, 2022. The trail was started in December, 2022. At the time of manuscript submission, patient recruitment for the trial is on-going. A total of 730 patients is expected to be enrolled in the intervention and control groups.

## Supplementary Information

**Additional file 1.**

## Data Availability

Not applicable.

## Abbreviations

DFS: Disease-free survival
XLJDOD: Xian-Lian-Jie-Du optimization decoction
CHM: Chinese herbal medicine
RR: Relapse rate
OS: Overall survival
QoL: Quality of life
IARC: International Agency for Research on Cancer
TCM: Traditional Chinese medicine
CAPOX: Capecitabine plus oxaliplatin
SPIRIT: Standard Protocol Items: Recommendations for Interventional Trials
CONSORT: Consolidated Standards of Reporting Trials
5-FU: 5-Fluorouracil
AJCC: American Joint Committee on Cancer
ECOG: Eastern Cooperative Oncology Group
SAE: Serious adverse event
CRF: Case report form
AE: Adverse event
IWRS: Interactive Web Response System
eCRF: Electronic case report form
EDC: Electronic Data Capture
SOP: Standard Operating Procedure
FAS: Full Analysis Set
PPS: Per-protocol Set
SS: Safety set

## References

[1] Sung H, Ferlay J, Siegel RL, Laversanne M, Soerjomataram I, Jemal A (2021). Global Cancer Statistics 2020: GLOBOCAN Estimates of Incidence and Mortality Worldwide for 36 Cancers in 185 Countries. CA Cancer J Clin.

[2] Sargent D, Sobrero A, Grothey A, O'Connell MJ, Buyse M, Andre T (2009). Evidence for cure by adjuvant therapy in colon cancer: observations based on individual patient data from 20,898 patients on 18 randomized trials. J Clin Oncol.

[3] Boland GM, Chang GJ, Haynes AB, Chiang YJ, Chagpar R, Xing Y (2013). Association between adherence to National Comprehensive Cancer Network treatment guidelines and improved survival in patients with colon cancer. Cancer.

[4] Feng RM, Zong YN, Cao SM, Xu RH (2019). Current cancer situation in China: good or bad news from the 2018 Global Cancer Statistics?. Cancer Commun (Lond).

[5] Fortea-Sanchis C, Forcadell-Comes E, Martinez-Ramos D, Escrig-Sos J (2019). Modelling the probability of erroneous negative lymph node staging in patients with colon cancer. Cancer Commun (Lond).

[6] Amin  MB, Greene  FL, Edge  SB, Compton  CC, Gershenwald  JE, Brookland  RK (2017). The Eighth Edition AJCC Cancer Staging Manual: Continuing to build a bridge from a population-based to a more "personalized" approach to cancer staging. CA Cancer J Clin.

[7] Grothey A, Sobrero AF, Shields AF, Yoshino T, Paul J, Taieb J (2018). Duration of adjuvant chemotherapy for stage iii colon cancer. N Engl J Med.

[8] Schmoll HJ, Tabernero J, Maroun J, de Braud F, Price T, Van Cutsem E (2015). Capecitabine plus oxaliplatin compared with fluorouracil/folinic acid as adjuvant therapy for stage iii colon cancer: final results of the NO16968 randomized controlled phase iii trial. J Clin Oncol.

[9] Labianca R, Beretta GD, Kildani B, Milesi L, Merlin F, Mosconi S (2010). Colon cancer. Crit Rev Oncol Hematol.

[10] Shah MA, Renfro LA, Allegra CJ, Andre T, de Gramont A, Schmoll HJ (2016). Impact of patient factors on recurrence risk and time dependency of oxaliplatin benefit in patients with colon cancer: analysis from modern-era adjuvant studies in the adjuvant colon cancer end points (ACCENT) database. J Clin Oncol.

[11] Kusumoto T, Ishiguro M, Nakatani E, Yoshida M, Inoue T, Nakamoto Y (2018). Updated 5-year survival and exploratory T x N subset analyses of ACTS-CC trial: a randomised controlled trial of S-1 versus tegafur-uracil/leucovorin as adjuvant chemotherapy for stage III colon cancer. ESMO Open.

[12] Smith TG, Troeschel AN, Castro KM, Arora NK, Stein K, Lipscomb J (2019). Perceptions of patients with breast and colon cancer of the management of cancer-related pain, fatigue, and emotional distress in community oncology. J Clin Oncol.

[13] Chen Q, Shu C, Laurence AD, Chen Y, Peng BG, Zhen ZJ (2018). Effect of Huaier granule on recurrence after curative resection of HCC: a multicentre, randomised clinical trial. Gut.

[14] Zhang S, Chen W, Wang Y, Wu J, Xu L, Yu Y (2021). Chinese herbal prescription Fu-Zheng-Qu-Xie prevents recurrence and metastasis of postoperative early-stage lung adenocarcinoma: a prospective cohort study followed with potential mechanism exploration. Oxid Med Cell Longev.

[15] Chen YX, Gao QY, Zou TH, Wang BM, Liu SD, Sheng JQ (2020). Berberine versus placebo for the prevention of recurrence of colorectal adenoma: a multicentre, double-blinded, randomised controlled study. Lancet Gastroenterol Hepatol.

[16] Xu Y, Mao JJ, Sun L, Yang L, Li J, Hao Y (2017). Association Between Use of Traditional Chinese Medicine Herbal Therapy and Survival Outcomes in Patients With Stage II and III Colorectal Cancer: A Multicenter Prospective Cohort Study. J Natl Cancer Inst Monogr.

[17] Yang Y, Ge J, Wu Y, Xu Y, Liang B, Luo L (2008). Cohort study on the effect of a combined treatment of traditional Chinese medicine and Western medicine on the relapse and metastasis of 222 patients with stage II and III colorectal cancer after radical operation. Chin J Integr Med.

[18] Tang M, Zhang W, Qin W, Zou C, Yan Y, He B (2022). Association between oral Chinese herbal medicine and recurrence and metastasis in patients with stages ii and iii colorectal cancer: a cohort study in China. Evidence-based complementary and alternative medicine : eCAM.

[19] Shi Q, Liu S, Li W, Zong S, Han S, Yang W (2017). Exploring the medication duration based on the effect of traditional Chinese medicine on postoperative stage I-III colorectal patients: a retrospective cohort study. Oncotarget.

[20] Jia R, Liu N, Cai G, Zhang Y, Xiao H, Zhou L (2021). Effect of PRM1201 combined with adjuvant chemotherapy on preventing recurrence and metastasis of stage iii colon cancer: a randomized, double-blind. Placebo-Controlled Clinical Trial Front Oncol.

[21] Cheng HB, Shen WX. [Relevance of cancer toxin pathogenesis theory with transformation of inflammation to carcinoma]. Zhongguo Zhong xi yi jie he za zhi Zhongguo Zhongxiyi jiehe zazhi = Chinese journal of integrated traditional and Western medicine. 2015;35(2):243–6. 25881473

[22] Wang J, Li L, Cheng H (2022). Clinical Study on Xian-Lia-Jie-Du decoction combined With XELOX regimen in the treatment of stage iv colorectal cancer. Acta Chin Med Pharmacol.

[23] Du Y, Wan H, Huang P, Yang J, He Y (2022). A critical review of Astragalus polysaccharides: from therapeutic mechanisms to pharmaceutics. Biomed Pharmacother.

[24] Jiang Y, Guo K, Wang P, Zhu Y, Huang J, Ruan S (2022). The antitumor properties of atractylenolides: molecular mechanisms and signaling pathways. Biomed Pharmacother.

[25] El-Saadony MT, Yang T, Korma SA, Sitohy M, Abd El-Mageed TA, Selim S (2022). Impacts of turmeric and its principal bioactive curcumin on human health: pharmaceutical, medicinal, and food applications: a comprehensive review. Front Nutr.

[26] Jia J, Li X, Ren X, Liu X, Wang Y, Dong Y (2021). Sparganii Rhizoma: a review of traditional clinical application, processing, phytochemistry, pharmacology, and toxicity. J Ethnopharmacol.

[27] Huang Y, Zhu J, Lin X, Hong Y, Feng Y, Shen L (2019). Potential of fatty oils from traditional chinese medicine in cancer therapy: a review for phytochemical, pharmacological and clinical studies. Am J Chin Med.

[28] He L, Zhong Z, Chen M, Liang Q, Wang Y, Tan W (2021). Current advances in coptidis rhizoma for gastrointestinal and other cancers. Front Pharmacol.

[29] Sun P, Zhao W, Wang Q, Chen L, Sun K, Zhan Z (2022). Chemical diversity, biological activities and Traditional uses of and important Chinese herb Sophora. Phytomedicine.

[30] Wen S, Zhang X, Wu Y, Yu S, Zhang W, Liu D (2022). Agrimonia pilosa Ledeb.: a review of its traditional uses, botany, phytochemistry, pharmacology, and toxicology. Heliyon.

[31] Chan AW, Tetzlaff JM, Gøtzsche PC, Altman DG, Mann H, Berlin JA (2013). SPIRIT 2013 explanation and elaboration: guidance for protocols of clinical trials. BMJ (Clinical research ed).

[32] Linde K, Brinkhaus B (2017). Randomized trials of Chinese herbal medicine: a new extension of the CONSORT statement. Ann Intern Med.

[33] Li Z, Shi Q, Liu M, Jia L, He B, Yang Y, et al. Validation and Application of the MD Anderson Symptom Inventory for Traditional Chinese Medicine (MDASI-TCM). J Natl Cancer Inst Monogr. 2017;2017(52). 10.1093/jncimonographs/lgx010 29140491

[34] Zheng R, Zhang S, Zeng H, Wang S, Sun K, Chen R (2022). Cancer incidence and mortality in China, 2016. J Natl Cancer Center.

[35] Chan AT, Ogino S, Fuchs CS (2009). Aspirin use and survival after diagnosis of colorectal cancer. JAMA.

[36] Rothwell P, Wilson M, Elwin C, Norrving B, Algra A, Warlow C (2010). Long-term effect of aspirin on colorectal cancer incidence and mortality: 20-year follow-up of five randomised trials. Lancet (London, England).

[37] Algra A, Rothwell P (2012). Effects of regular aspirin on long-term cancer incidence and metastasis: a systematic comparison of evidence from observational studies versus randomised trials. Lancet Oncol.

[38] Rothwell P, Wilson M, Price J, Belch J, Meade T, Mehta Z (2012). Effect of daily aspirin on risk of cancer metastasis: a study of incident cancers during randomised controlled trials. Lancet (London, England).

[39] Domingo E, Church D, Sieber O, Ramamoorthy R, Yanagisawa Y, Johnstone E (2013). Evaluation of PIK3CA mutation as a predictor of benefit from nonsteroidal anti-inflammatory drug therapy in colorectal cancer. J Clin Oncol.

[40] Meyerhardt JA, Shi Q, Fuchs CS, Meyer J, Niedzwiecki D, Zemla T (2021). Effect of celecoxib vs placebo added to standard adjuvant therapy on disease-free survival among patients with stage iii colon cancer: the CALGB/SWOG 80702 (Alliance) randomized clinical trial. JAMA.

[41] Morales-Oyarvide V, Meyerhardt JA, Ng K (2016). Vitamin D and physical activity in patients with colorectal cancer: epidemiological evidence and therapeutic implications. Cancer journal (Sudbury, Mass).

[42] Fuchs M, Yuan C, Sato K, Niedzwiecki D, Ye X, Saltz L (2017). Predicted vitamin D status and colon cancer recurrence and mortality in CALGB 89803 (Alliance). Ann Oncol.

[43] Urashima M, Ohdaira H, Akutsu T, Okada S, Yoshida M, Kitajima M (2019). Effect of Vitamin D supplementation on relapse-free survival among patients with digestive tract cancers: The AMATERASU randomized clinical trial. JAMA.

[44] Mc Menamin ÚC, Murray LJ, Hughes CM, Cardwell CR (2016). Metformin use and survival after colorectal cancer: A population-based cohort study. Int J Cancer.

[45] Du L, Wang M, Kang Y, Li B, Guo M, Cheng Z (2017). Prognostic role of metformin intake in diabetic patients with colorectal cancer: an updated qualitative evidence of cohort studies. Oncotarget.

[46] Vernieri C, Galli F, Ferrari L, Marchetti P, Lonardi S, Maiello E (2019). Impact of metformin use and diabetic status during adjuvant fluoropyrimidine-oxaliplatin chemotherapy on the outcome of patients with resected colon cancer: a TOSCA Study subanalysis. Oncologist.

[47] Christou N, Bergen E, Canton C, Le Malicot K, Di Bartolomeo M, Galli F (2022). Impact of diabetes and metformin use on recurrence and outcome in stage II-III colon cancer patients-A pooled analysis of three adjuvant trials. Eur J Cancer (Oxford, England : 1990).

[48] McQuade JL, Meng Z, Chen Z, Wei Q, Zhang Y, Bei W (2012). Utilization of and attitudes towards traditional Chinese medicine therapies in a Chinese cancer hospital: a survey of patients and physicians. Evidence-based complementary and alternative medicine : eCAM.

[49] Chen G, Qiao TT, Ding H, Li CX, Zheng HL, Chen XL, et al. Use of Chinese herbal medicine therapies in comprehensive hospitals in central China: a parallel survey in cancer patients and clinicians. Journal of Huazhong University of Science and Technology Medical sciences = Hua zhong ke ji da xue xue bao Yi xue Ying De wen ban = Huazhong keji daxue xuebao Yixue Yingdewen ban. 2015;35(6):808–14. 10.1007/s11596-015-1511-5 26670429

[50] Lv J, Jia Y, Li J, Kuai W, Li Y, Guo F (2019). Gegen Qinlian decoction enhances the effect of PD-1 blockade in colorectal cancer with microsatellite stability by remodelling the gut microbiota and the tumour microenvironment. Cell Death Dis.

[51] Huang S, Peng W, Mao D, Zhang S, Xu P, Yi P (2019). Kangai Injection, a traditional Chinese medicine, improves efficacy and reduces toxicity of chemotherapy in advanced colorectal cancer patients: a systematic review and meta-analysis. Evidence-based complementary and alternative medicine : eCAM.

[52] Sun L, Yan Y, Chen D, Yang Y (2020). Quxie capsule modulating gut microbiome and its association with t cell regulation in patients with metastatic colorectal cancer: result from a randomized controlled clinical trial. Integr Cancer Ther.

[53] Dai Y, Wang H, Sun R, Diao J, Ma Y, Shao M (2022). Modified Shenlingbaizhu decoction represses the pluripotency of colorectal cancer stem cells by inhibiting TGF-β mediated EMT program. Phytomedicine.

[54] Yu C, Chen T, Lu S, Hu W, Zhang Q, Tan J (2022). Identification of significant modules and targets of Xian-Lian-Jie-Du decoction based on the analysis of transcriptomics, proteomics and single-cell transcriptomics in colorectal tumor. J Inflamm Res.

[55] Zhang Q, Cheng H, Yu C, Shen W, Xu C, Tan J (2022). Effect of Xianlian Jiedu formula on intestinal cell types in colorectalcancer model mice with damp-heat stasis toxin pattern. J Tradit Chin Med.

[56] Chen T, Yu C, Xu H, Cheng H, Shen W, Tan J (2022). Transcriptome analysis of Xianlian Jiedu prescription in intervention of colorectalcarcinoma due to dampness, heat, stasis, and toxin in mice. Chin J Exp Tradit Med Formulae.

[57] Shao C, Zuo Q, Lin J, Yu RJ, Fu Y, Xiao M (2019). Effect of Chinese herbal medicine on the survival of colorectal cancer patients with liver-limited metastases: a retrospective cohort study, 2008 to 2017. Integr Cancer Ther.

